# Supplementary material for: Facebook addiction and its related factors among medical students; a cross- sectional study in Bangladesh
Source: PLOS Glob Public Health. 2023 Feb 22;3(2):e0001597. doi: 10.1371/journal.pgph.0001597 (PMC10021403; doi:10.1371/journal.pgph.0001597)
Supplement: Attachment — Submitted filename: Response to Reviewers.docx [file pgph.0001597.s001.docx]

**Response to reviewers**

**Journal Requirements**

1. Please amend your detailed Financial Disclosure statement. This is published with the article. It must therefore be completed in full sentences and contain the exact wording you wish to be published.

a. Please clarify all sources of funding (financial or material support) for your study. List the grants (with grant number) or organizations (with url) that supported your study, including funding received from your institution. 
b. State the initials, alongside each funding source, of each author to receive each grant.
c. State what role the funders took in the study. If the funders had no role in your study, please state: “The funders had no role in study design, data collection and analysis, decision to publish, or preparation of the manuscript.”

Done as suggested>>

“This research project was funded by Bangladesh Medical Research Council BMRC (BMRC/HPNSP-Research 2021 -2022; 1471). The sponsor helped by paying the authors honoraria, printing the questionnaire, and purchasing stationery, but they had no further involvement in the study's planning, data collecting, analysis, publication decision, or manuscript preparation.” Line # 556-560

2. Please note that your Data Availability Statement is currently missing [the repository name. If your manuscript is accepted for publication, you will be asked to provide these details on a very short timeline. We therefore suggest that you provide this information now, though we will not hold up the peer review process if you are unable.

Done as suggested>>

Data is freely available at the following website. “https://figshare.com/articles/dataset/Facebook_addiction_and_its_related_factors_in_medical_students_Bangladesh_context/20377236”

3. We do not publish any copyright or trademark symbols that usually accompany proprietary names, eg (R), (C), or TM  (e.g. next to drug or reagent names). Please remove all instances of trademark/copyright symbols throughout the text, including © on line 500.

Done>>

“©” removed

“Andreassen C S. Development of a Facebook addiction scale. Psychological Reports, 2012, 110, 2, 501-517. Psychological Reports 2012.

DOI 10.2466/02.09.18.PR0.110.2.501-517 ISSN 0033-2941

Line # 708-710

4. Please provide separate figure files in .tif or .eps format and remove the embedded figures from the manuscript file.

Done>> as suggested [tiff file]

Reviewer #1: First of all, the title of the study should include the study design which is a "cross-sectional survey". Second, the abstract should comprise the main statistical tests that have been used to identify the predictors(i.e. OR). Third, I would rather that the study follows a certain model to justify the section of the potential predictors. For instance, Anderson Behavioral Model. Furthermore, the study has not identified whether the respondents had consented and what type of consent was used ( written or verbal). Moreover, the study was not clearly identified the language used in data collection (i.e. English or any other language).
There were nineteen independent variables that entered the analysis using Binary Logistic Regression (BLR), however, the study had not provided a robust justification for compromising only 19 out of the rest of the independent variables.
The discussion part of the study did not make clear what this research adds to existing research. Furthermore, the study did not describe how this research advances our understanding or how it may inspire future applications. For instance, the study declared that "Our data showed that factors such as personal income, living with their family, perceived positive, family relationships, poor academic performance, moderate anxiety, moderately severe, depression, Internet addiction, and moderate to high perceived stress were statistically, significantly associated with Facebook addiction", however, it had not provided a possible interpretation.
The implications of the study should specify the type of intervention that is recommended to tackle the problem of Facebook addiction(e.g. an educational or behavioral intervention) and the target population based on the risk factors identified in this study.

**Response to reviewer #1**

Study design included in the title of the study

“Facebook addiction and its related factors among medical students; a cross-sectional study in Bangladesh” Line # 1,2

The statistical test findings (Odds ratio with 95% CI) were incorporated into a revised version of the abstract. Line # 23-46

In this study, no particular behavioral model was used to evaluate the determinants of Facebook addiction.

Line 311-321, Line # 382-391

Concerning informed consent, a specific statement was included in the manuscript's “Declaration” section under "Ethical approval and consent to participate." Line # 532-537

The language used in data collecting was discussed in length in the section on “Data collection.” Line # 185-190

A few changes were made in the Discussion section in response to the reviewer's inquiry. Line # 489-494

The conclusion part was substantially updated, and specific measures [interventions] were recommended. Line # 511-527

Reviewer #2: well planned executed and presented study.
intorduction - impact of overall social media on medical students can be added.
need to add more information about questionnaires used. validation, reliability and scoring need to be added.

**Response to reviewer #2**

No additional information was added in introduction section.

Detailed information about questionnaires e.g. validation, reliability and scoring were added.

Internet Addiction Line # 219-228 , Facebook Addiction Line # 241-248, Perceived stress Line # 263-272 , Depression Line # 274-283, Anxiety Line # 285-294, Sleep quality Line # 300-308
