# Supplementary material for: Facebook addiction and its related factors among medical students; a cross- sectional study in Bangladesh
Source: PLOS Glob Public Health. 2023 Feb 22;3(2):e0001597. doi: 10.1371/journal.pgph.0001597 (PMC10021403; doi:10.1371/journal.pgph.0001597)
Supplement: Attachment — Submitted filename: Response to reviewers.docx [file pgph.0001597.s002.docx]

**Response to reviewers**

**Comments**

Reviewer #1: Please check the following points: Prior to running the analysis for the BLR, important underlying assumptions should be checked such as sample size and multicollinearity.

**Response**

**Line 353-359**

The model was preliminarily checked for sampling adequacy and multicolinearity. According to a study, in observational studies with large populations that employ logistic regression in the analysis, a minimum sample size of 500 must be taken to produce statistics that correctly reflect the parameters.^46^We assessed multicollinearity by examining correlation indices and variance inflation factor (VIF) values. The magnitudes of the calculated coefficients of the predictor variables were less than.80, indicating that they were not highly correlated. The VIF values of the predictor variables, however, were likewise observed to be below 5.00.

**Comments**

Please specify the time of the discussion session for the participants

Please specify the research team

**Response**

**Line 136-141**

The research team, which consisted of the lead author, co-authors, supervisors, and data collectors, organized a discussion session on the study procedure (Aim of the Study and Instruction Manual for the Questionnaire) for each year's students with the assistance of the Community Medicine Department of the respective medical college. The explanation and discussion session lasted around 1.30 hours (one hour for presentation and thirty minutes for questions and answers).

**Comments**

Please provided the consent form and specify its type (verbal, written...) Please specify how participant privacy and confidentiality were assured?

**Response**

**Line 508-513**

We then obtained their written informed consent using a separate consent form attached to the main questionnaire. Respondents were given the assurance that any information they provided, such as their names or any other information that could be used to identify them, would be kept confidential and anonymous by substituting codes for participant identifiers and storing data in a locked cabinet, and that the anonymized data and or study results would only be disseminated and published in the public interest.

**Comments**

The section of predicators variables was based on the literature review, please sure that you provided the appropriate citations in every section of the manuscript.

**Response**

Done as suggested

**Comments**

Please specify if all study variables were entered into the BLR model (All entered) or any other approach

**Response**

**Line 344-346**

Binary logistic regression employing the "enter" strategy was used to explore the effects of a variety of demographic, psychological, and behavioral traits on the chance that respondents had a Facebook addiction

**Comments**

Please make the appropriate editing and grammars checking for all the manuscript

**Response**

**Done as suggested**
